# A comparative policy analysis of the adoption and implementation of sugar-sweetened beverage taxes (2016–19) in 16 countries

**DOI:** 10.1093/heapol/czac004

**Published:** 2022-03-04

**Authors:** Georgina Mulcahy, Tara Boelsen-Robinson, Ashleigh Chanel Hart, Maria Amalia Pesantes, Mohd Jamil Sameeha, Sirinya Phulkerd, Reem F Alsukait, Anne Marie Thow

**Affiliations:** Menzies Centre for Health Policy, Sydney School of Public Health, University of Sydney, Australia; Menzies Centre for Health Policy, Sydney School of Public Health, University of Sydney, Australia; Global Obesity Centre (GLOBE), Institute for Health Transformation, Deakin University, Locked Bag 20000, Geelong, Victoria 3220, Australia; The George Institute for Global Health, Level 5, 1 King Street, Newtown, New South Wales 2042, Australia; CRONICAS Centro de Excelencia en Enfermedades Crónicas, Universidad Peruana Cayetano, HerediaAv. Armendáriz 445, Miraflores Lima 18, Peru; Department of Anthropology and Archaeology, Dickinson College, 28N College Street, Carlisle, PA 17013, USA; Centre for Community Health Studies (ReaCH), Faculty of Health Sciences, Universiti Kebangsaan Malaysia (UKM), Jalan Raja Muda Abdul Aziz, 50300, Kuala Lumpur, Malaysia; Institute for Population and Social Research, Mahidol University, Phutthamonthon, Nakhon Pathom 73170, Thailand; Department of Community Health Sciences, College of Applied Medical Sciences, King Saud University, King Saud University, Riyadh 11362, Saudi Arabia; Menzies Centre for Health Policy, Sydney School of Public Health, University of Sydney, Australia

**Keywords:** Public health, nutrition, policy analysis, food policy, policy research, policy implementation

## Abstract

Taxes on sugar-sweetened beverages (SSBs) are recommended as part of comprehensive policy action to prevent diet-related non-communicable diseases (NCDs), but have been adopted by only one quarter of World Health Organization (WHO) Member States. This paper presents a comparative policy analysis of recent SSB taxes (2016–19) in 16 countries. This study aimed to analyse the characteristics and patterns of factors influencing adoption and implementation of SSB taxes and policy learning between countries, to draw lessons for future SSB taxes. The data collection and analysis were informed by an analytical framework that drew on ‘diffusion of innovation’ and theories of policy learning. Qualitative data were collected from policy documents and media, in addition to national statistics. Qualitative data were thematically analysed and a narrative synthesis approach was used for integrated case study analysis. We found adaptation and heterogeneity in the approaches used for SSB taxation with a majority of countries adopting excise taxes, and consistent health framing in media and policy documents. Common public frames supporting the taxes included reducing obesity/NCDs and raising revenue (government actors) and subsequent health system savings (non-government actors). Opposing frames focused on regressivity and incoherence with other economic policy (government actors) and posited that taxes have limited health benefits and negative economic impacts on the food industry (industry). Evident ‘diffusion networks’ included the WHO, predominantly in middle-income countries, and some regional economic bodies. We found indications of policy learning in the form of reference to other countries’ taxes, particularly countries with membership in the same economic bodies and with shared borders. The study suggests that adoption of SSB taxation could be enhanced through strategic engagement by health actors with the policy-making process, consideration of the economic context, use of consistent health frames by cross-sector coalitions, and robust evaluation and reporting of SSB taxation.

**Key messages**

- Analysing characteristics and patterns of previous sugar-sweetened beverage (SSB) tax adoption may inform other countries’ approach
- Consistent public frames were identified both supporting (NCD reduction and health system savings) and opposing (regressivity and economic impacts on food industry) SSB tax adoption
- Policy learning was enhanced through diffusion networks such as World Health Organization (WHO) and regional economic bodies
- Wide reporting of robust evaluations of previously adopted SSB taxes strengthened the evidence of effectiveness for adoption

## Introduction

Non-communicable diseases (NCDs) are the cause of 71% of all deaths and over 80% of premature deaths globally each year (WHO 2018a). NCDs also have significant economic impacts on health systems and human capital, due to high health-care costs and reduced workforce productivity (Bloom *et al.*, 2011). The causes of NCDs are multifactorial, including unhealthy diets, tobacco, alcohol, insufficient physical activity, and air pollution (WHO, 2018a,b). Sugar-sweetened beverage (SSB)^1^ consumption has been identified as an independent risk factor for NCDs (WHO, 2013). There is strong and consistent evidence of a dose–response relationship between SSB consumption and adverse cardiovascular and metabolic health, resulting in obesity, cardiovascular disease and type-2 diabetes mellitus (Deshpande *et al.*, 2017; YU *et al.*, 2018; Malik and Hu 2019).

Taxes on SSBs are recommended by the World Health Organization (WHO) and other global bodies as part of comprehensive policy action to prevent diet-related NCDs (WHO, 2013; FAO & WHO, 2014; World Bank, 2020). SSB taxes increase prices of SSBs, which decreases consumer demand (Cawley *et al.*, 2019; Alsukait *et al.*, 2020b; Phulkerd *et al.*, 2020). An increased tax burden also incentivizes manufacturers to reformulate beverages to contain less sugar (WHO, 2016). Reduced SSB and sugar consumption has flow-on effects to decreased mortality rates from NCDs, decreased health system expenditure and increased workforce productivity (World Health Organization Regional Office for Europe, 2015; Saxena *et al.*, 2019a,b).

SSB taxation has been shown to be a feasible, acceptable and effective policy option for addressing obesity and NCDs; however, only around one quarter of WHO Member States have adopted SSB taxes following global recommendations (WCRF, 2020). Challenges influencing the adoption of SSB taxes include the cross-sectoral nature of SSB taxation, as health, finance and other sectors of government have vastly different priorities, as well as a lack of political will and conflicting perceptions of effectiveness (Graça *et al.*, 2018; Thow *et al.*, 2018a; Onagan *et al.*, 2019; Alsukait *et al.*, 2020a). These challenges suggest a need to support governments to adopt and implement globally recommended ‘best practice’ interventions such as SSB taxes. Such global recommendations are adopted (or resisted) and adapted through complex policy and political processes. An analysis of the diffusion of global policy recommendations at the national level can thus offer insights into factors and strategic actions by the health sector that might foster further uptake of these recommendations.

Previous research has indicated that there have been shared experiences between countries in SSB tax adoption and implementation and that there is potential for policy learning (Carriedo *et al.*, 2021; Thow *et al.*, 2021a). Studies have identified a need to engage with non-governmental stakeholders, including industry and civil society, whose power and interests affect the agenda setting and decision-making processes (Thow *et al.*, 2011; James *et al.*, 2020; Alsukait *et al.*, 2020a). A key facet is the framing of SSB taxes by governments as both a health promotion measure and a fiscal imperative (Hagenaars *et al.*, 2017). In a policy context, framing refers to the way in which policy issues and policy measures are portrayed and understood; framing occurs with respect to both the policy ‘problem’ and ‘solution’ (Kingdon, 1984). Analyses of health policy processes more broadly have indicated that adoption of evidence-based policy can be fostered through strategic health sector framing of the health problem and policy solution, developing strong actor networks, and engaging with paradigms and ideas in relevant sectors (Shiffman and Smith 2007; Shiffman, 2016; Wickramasinghe *et al.*, 2018).

This study builds on existing literature by examining more recent adoption of SSBs (2016–19), as uptake of this global recommendation has begun to diffuse more widely. The aim of this study was to analyse the characteristics and patterns of potential factors influencing adoption and implementation of SSB taxes in 16 countries, as well as indications of policy learning that have occurred between the countries, in order to draw lessons for future SSB taxes.

## Methods

### Study design

We conducted a policy analysis focused on potential influences on the adoption and implementation of recent (2016–19) SSB taxes in 16 case study countries. These policies followed global recommendations for national-level policy action, and we thus considered them as ‘diffusion’ of (policy) innovation (Rogers, 2003). We utilized case study research methods to guide case selection, data collection, cross-case synthesis and analysis (Yin, 2003). Our study drew on documentary data and used qualitative analysis and narrative synthesis to answer the following questions: What are the characteristics and patterns, across the elements of the framework, relevant to SSB tax adoption? How has the innovation (SSB tax) been adapted across different contexts? What are the lessons for future adopters of SSB taxes?

### Study framework

In adapting diffusion of innovation theory to a policy context, we observed that the uptake and diffusion of policy is influenced by, amongst other things, a process of policy learning between countries (Rose, 1993; Stone, 2004). We thus adapted diffusion of innovation theory (Rogers, 2003) for our study framework (Table 1) by augmenting it with insights from theories of policy learning (Rose, 1993; Stone, 2004; 2019), similar to the approach taken by Linos (2013). Table 1 presents key theoretical concepts and characteristics of each element of the analytical framework and indicators (specific measures that speak to the elements of the framework) for data collection and analysis that were derived with reference to previous literature, particularly Hagenaars *et al.*, (2017). Indicators related to framing of the policy problem and taxation as a ‘policy solution’ were derived from textual representations regarding the context of, reason for, and implications of, SSB taxes in policies and the media. For the indicators relevant to mechanisms of engagement in the policy process, we also drew on the ‘framework for categorizing the corporate political activity of the food industry with respect to public health’, which provided a clear categorization of mechanisms (Mialon *et al.*, 2015). Although this framework focuses on food industry (corporate) political activity, we observed that it spanned policy change broadly [i.e. mechanisms to effect (or prevent) food and nutrition policy change], and we thus considered the way these strategies were used by all actors in the policy process.

**Table 1.** Theoretical Framework

Element of Diffusion of Innovation Theory	Theoretical characteristic (also drawing on policy learning theory)	Indicator	Data source
The innovation An innovation is an idea, practice or object that individuals, groups or other units of adoption perceive as new (Rogers, 2003). In this study, the innovation are SSB taxes.	Setting of a policy instrument (Hall, 1993)	Tax name Tax type Tax base Tax rate/s	Policy documents Government websites
	Political feasibility and desirability (Rose, 1993) Relative advantage (Rogers, 2003)	Framing of the SSB tax Framing of other policy options	Newspapers and news websites Policy documents Government websites
	Compatibility (Rogers, 2003) Context (Rose, 1993)	Existing health-related taxes	Policy documents Government websites
Diffusion networks Diffusion networks are means to convey information on an innovation, often through opinion leadership of communication networks (Rogers, 2003). In this study, diffusion networks include countries, multilateral institutions and regional economic bodies.	Non-state actors and international organizations (Stone, 2004, Stone, 2019)	Reference to health multilateral institutions Reference to economic multilateral institutions Reference to other multilateral institutions	Newspapers and news websites
	Proximity (Rose, 1993) Homophily (Rogers, 2003)	Regionality	WHO regions (Regional Office for the Americas of the World Health Organization, 2021; WHO Regional Office for Africa, 2021; WHO Regional Office for Europe, 2021; WHO Regional Office for the Eastern Mediterranean, 2021; World Health Organization Western Pacific Region, 2021; WHO Regional Office for South East Asia, 2021)
		Language	CIA World Factbook (Central Intelligence Agency, 2021)
	Membership in domestic and international policy communities (Stone, 2019)	Regional economic bodies	CIA World Factbook (Central Intelligence Agency, 2021)
	Observability (Rogers, 2003)	Reporting of other countries’ SSB taxes	Newspapers and news websites
Change agents Change agents are individuals or groups who take actions with a coherent objective, to bring about change with an intended outcome (Rogers, 2003). In this study, change agents are state, non-state and transnational actors who are actively ‘for’ or ‘against’ the SSB tax (Stone *et al.*, 2019; Kingdon, 1984).	Non-state actors and international organizations; proximity to decision-makers; access to political stakeholders (Stone, 2004; Stone, 2019)	Name and type of actor/s and/or advocacy coalition/s	Newspapers and news websites
	Framework for categorizing the corporate political activity of the food industry with respect to public health (Mialon *et al.*, 2015)	Mechanism for engagement with the policy process	
Innovating countries An innovator is an individual or group that can adopt an innovation (Rogers, 2003). In this study, innovators are ‘early majority’ and ‘early adopter’ countries who have adopted SSB taxes.	Access to decision makers (Stone, 2019) Functional and/or physical proximity from decision makers (Rogers, 2003)	Public consultation on issue/innovation Political participation	Newspapers and news websites Government websites EIU Democracy Index 2019 (The Economist Intelligence Unit, 2020)
	Values (Rogers, 2003) Political values (Rose, 1993) Context (Rose, 1993)	Framing of health by government Fiscal priorities of government Presence of tax reform	Policy documents—national and health policies Policy documents—national policies Newspapers and news websites Government websites
		Governing regime	Regimes of the World, v-Dem (Coppedge *et al.*, 2020a,b)
		Government health expenditure	World Bank (The World Bank, 2021b)
	Socio-economic status (Rogers, 2003) Resources (Rose, 1993)	Income group Gross National Income (GNI) per capita	World Bank (The World Bank, 2021e) World Bank (The World Bank, 2021d)
		Inequality—Gini index	World Bank (The World Bank, 2021c)
	Political values (Rose, 1993)	Framing of the problem	Newspapers and news websites
	Health context	Prevalence of NCDs and SSB consumption	World Bank (The World Bank, 2021a) Global Health Observatory (The Global Health Observatory, 2021a,b) Global Dietary Database (Tufts Friedman School of Nutrition Science and Policy, 2019)

### Case study selection

We based our case study selection on the adopter categories identified by Rogers (2003), and specifically the ‘early majority’ adopters of SSB taxation, as the 16th–50th percentile of WHO Member States to adopt an SSB tax (Rogers, 2003). These follow ‘innovators’ (the first 2.5%), e.g., Pacific Island countries that adopted SSB taxation in the 2000s (Thow *et al.*, 2011), and ‘early adopters’ (2.5–16%), e.g., Mexico. These more recent adopters of SSBs have been less researched to date, and they may be more relevant to future policy learning by countries that have been slower to adopt SSB taxes, as they are likely to have similarities with ‘late majority adopters’, who have yet to adopt SSB taxes (Rogers, 2003). We used data from the World Cancer Research Fund NOURISHING database (WCRF, 2020) to create a timeline of adopters and determined the first ‘early majority’ country was the 31st country to adopt an SSB tax (i.e. the 16th percentile out of 194 WHO Member States). This was a tax adopted during 2017; however, as the database variously reported adoption or implementation dates (which can differ markedly), we determined our case study countries as those that were reported as having implemented SSB taxes from the beginning of 2017 onwards.^2^ Sixteen countries were included in the sample, in February 2020.

### Data collection

We collected documentary data from three sources: policy documents, national statistics and media. These sources are detailed in Table 1; they were identified based on previous research as providing complementary perspectives on the key elements of the study framework (Hagenaars *et al.*, 2017; Rowbotham *et al.*, 2019). The time period for data collection was the year of adoption of the tax for the policy documents (i.e. the current policy at that time) and for national statistics, and the five-year period surrounding the adoption of the tax (3 years prior to year of adoption, the year of adoption and 1 year post-adoption).

To maximize the quality of the data, the research team conducted the initial data collection in the language in which the documents were originally written where possible: Arabic, Bahasa Melayu, English, French, Spanish and Thai. This approach reflects our priority to ensure adequate representation of middle-income countries (MICs), as although these countries have often been leaders in SSB taxation and other health policy interventions, they tend to be underrepresented in international literature.

#### Policy documents

We drew on policy documents and government websites (namely health, finance and revenue departments) from the case study countries, to inform the indicators related to the policy instrument and presence of existing health-related taxes (‘innovation’), as well as framing of the government’s health and fiscal priorities (Table 1). We conducted targeted searches via Google for government websites and specific policy documents current at the time of adoption of the tax. Search terms included the country name, policy [or strategy, plan] and key terms related to the type of policy sought (e.g. national development and health policy).

#### National statistics

We identified data sources to document key indicators for the ‘innovating countries’ (Table 1). The selection of these sources was informed by the data sources identified for the policy context by Hagenaars *et al.* (2017) and the explanatory variables by Allen *et al.* (2020).

#### Print and online media

We drew on data from articles in newspapers and news websites, available through an online database (Factiva), to inform indicators related to the framing of the ‘innovation’, the framing of the problem from the perspective of the innovating countries, as well as the presence of ‘diffusion networks’ and ‘change agents’ (Table 1). The media searching, sampling and data collection were guided by Buckton *et al.* (2018). Details of the media search and sampling process are outlined in Supplementary Material #1.

### Preliminary data analysis

#### Policy documents

We created typologies for the policy instrument indicators, within the ‘innovation’, based on Chriqui *et al.* (2013). Themes were inductively determined for the indicators regarding the innovating countries, including framing of health and economic priorities of government, from the English language data of eight countries. A template of the typologies and themes was developed for the deductive analysis of the policy documents to be conducted in Arabic, Thai, Spanish and Bahasa Melayu by four researchers (RFA, SP, MAP, MJS) in eight case study countries.

#### Print and online media

The coding and preliminary analysis of the media data was conducted using NVivo 12™ by inductively developing themes and determining typologies for ‘mechanisms of engagement’ by policy actors, which were adapted from the framework by Mialon *et al.* (2015). The process of analysis and codebook are outlined in Supplementary Material #1.

### Integrated case study analysis

Data for all indicators and countries were entered into a custom matrix (Microsoft Excel™) to enable cross-country and cross-indicator analysis. Two researchers (GM and AMT) analysed the data for each country, across the four elements of the theoretical framework, to develop case descriptions. Following this, they used the full data matrix (Microsoft Excel™) to conduct a cross-case analysis. Inductive explanation-building was undertaken by determining patterns between indicators, within and across the four elements of the theoretical framework (Table 1; Yin, 2003). Throughout the analysis process, meetings were conducted with all researchers to review the preliminary findings and to conduct further cross-case analysis and inductive explanation-building using the data matrix.

## Results

### Overview of countries included in the study

**Figure 1.**
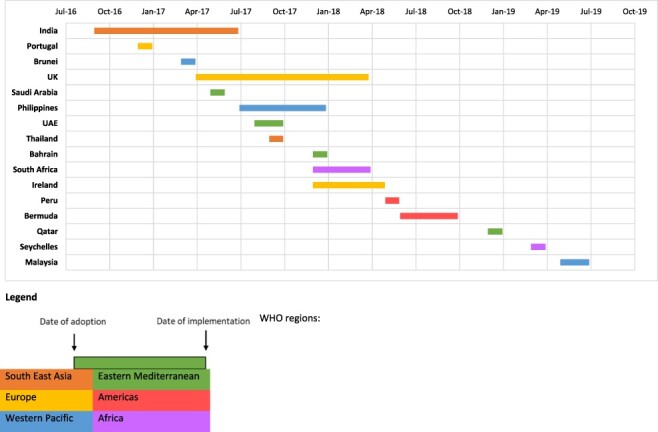
Timeline of adoption to implementation of sugar-sweetened beverage taxes, ordered by date of adoption

**Figure 2.**
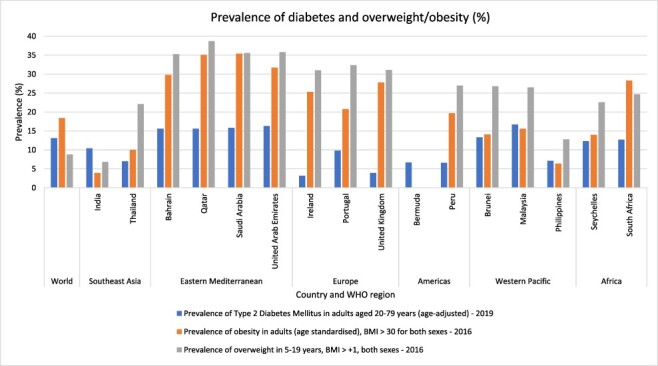
Prevalence of type 2 diabetes mellitus, (adult) obesity and (child) overweight

Source: Diabetes (The World Bank, 2021a); Overweight and obesity (The Global Health Observatory, 2021a,b).

**Figure 3.**
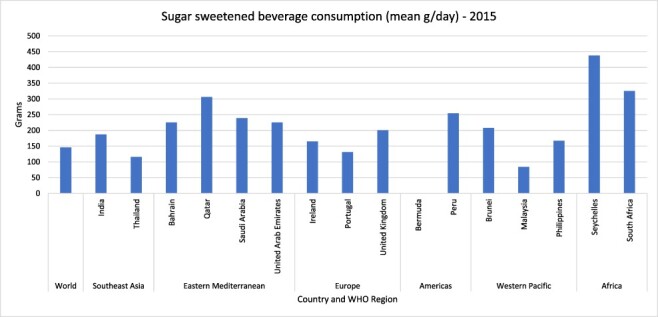
Sugar-sweetened beverage consumption

Source: Global Dietary Database (Tufts Friedman School of Nutrition Science and Policy, 2019).

The 16 countries in this study adopted SSB taxes between September 2016 and May 2019, which were subsequently implemented between July 2017 and July 2019 (Figure 1). The countries were located across all six of the WHO regions (Figure 1, Table 2). There were 10 high-income countries (HICs) and six MICs (Table 2). All countries had a documented burden of diet-related NCDs, including type 2 diabetes, overweight and obesity (Figure 2). The prevalence varied across the countries, with the Eastern Mediterranean region countries having the highest prevalence of diet-related NCDs (Figure 2). Consumption of SSBs prior to adoption of the taxes (2015) ranged from around 80 g/capita/day in Malaysia to over 400 g/capita/day in Seychelles (Figure 3). Public consultation for the tax occurred in six countries (Bermuda, India, Ireland, Philippines, South Africa and UK) with high levels of political participation and governing regimes categorized as liberal or electoral democracies, except the Philippines (Table 2).

**Table 2.** Overview of case study countries

Country	Region	Official language/s	Regional economic body membership	Income Group, 2021	GNI per capita, 2019	Inequality (Gini index)	Political participation score, 2019	Type of governing regime, 2019	Government health expenditure, 2017
Source	WHO regions^a^	CIA World Factbook^b^	CIA World Factbook^b^	World Bank income groups^c^	World Bank^d^	World Bank^e^	EIU Democracy Index^f^	Regimes of the World, v-Dem^g^	World Bank^h^
Bahrain	Eastern Mediterranean	Arabic	Gulf Cooperation Council (GCC)	High-income country	21 890	N/A	2.78	Closed autocracy	58%
Bermuda	Americas	English, Portuguese	N/A	High-income country	106 140	N/A	N/A	N/A	N/A
Brunei	Western Pacific	Malay (Bahasa Melayu)	Association of Southeast Asian Nations (ASEAN), ASEAN Economic Community (AEC), Asia-Pacific Economic Cooperation (APEC)	High-income country	31 020	N/A	N/A	N/A	95%
India	South East Asia	Hindi, English and 22 other officially recognised languages.	BRICS (i.e. Brazil, Russia, India, China, South Africa), South Asian Association for Regional Cooperation (SAARC), South Asian Free Trade Area (SAFTA)	Lower-middle income country	2020	36 (2011)	6.67	Electoral democracy	27%
Ireland	Europe	English Irish	European Union (EU)	High-income country	59 770	33 (2016)	8.33	Liberal democracy	73%
Malaysia	Western Pacific	Malay (Bahasa Melayu)	Association of Southeast Asian Nations (ASEAN), ASEAN Economic Community (AEC), Asia-Pacific Economic Cooperation (APEC)	Upper-middle income country	10 460	41 (2015)	6.67	Electoral autocracy	51%
Peru	Americas	Spanish, Quechua, Aymara	Andean Community, Asia-Pacific Economic Cooperation (APEC), Free Trade Area of the Americas (FTAA), Pacific Alliance, Union of South American Nations (UNASUR)	Upper-middle income country	6530	43 (2018)	5.56	Electoral democracy	61%
Philippines	Western Pacific	Filipino, English	Association of Southeast Asian Nations (ASEAN), ASEAN Economic Community (AEC), Asia-Pacific Economic Cooperation (APEC)	Lower-middle income country	3830	44 (2015)	7.22	Electoral autocracy	32%
Portugal	Europe	Portuguese Mirandese	European Union (EU)	High-income country	21 680	34 (2017)	6.61	Liberal democracy	66%
Qatar	Eastern Mediterranean	Arabic	Gulf Cooperation Council (GCC)	High-income country	61 190	N/A	2.22	Closed autocracy	81%
Saudi Arabia	Eastern Mediterranean	Arabic	Gulf Cooperation Council (GCC)	High-income country	21 540	N/A	2.22	Closed autocracy	64%
Seychelles	Africa	Seychellois Creole, English, French	Common Market for Eastern and Southern Africa (COMESA), Southern African Development Community (SADC)	High-income country	15 600	47 (2013)	N/A	Electoral democracy	73%
South Africa	Africa	isiZulu, isiXhosa, Afrikaans, Sepedi, Setswana, English, Sesotho, Xitsonga, siSwati, Tshivenda, isiNdebele	BRICS (i.e. Brazil, Russia, India, China, South Africa), Southern African Development Community (SADC)	Upper-middle income country	5750	63 (2014)	8.33	Electoral democracy	54%
Thailand	Southeast Asia	Thai	Association of Southeast Asian Nations (ASEAN), ASEAN Economic Community (AEC), Asia-Pacific Economic Cooperation (APEC)	Upper-middle income country	6610	36 (2018)	5.00	Closed autocracy	76%
United Arab Emirates	Eastern Mediterranean	Arabic	Gulf Cooperation Council (GCC)	High-income country	41 010	33 (2014)	2.22	Closed autocracy	72%
United Kingdom	Europe	English	European Union (at the time of adoption)	High-income country	41 340	35 (2016)	8.89	Liberal democracy	79%

Notes and Sources:

a Regional Office for the Americas of the World Health Organization (2021), WHO Regional Office for Africa (2021), WHO Regional Office for Europe (2021), WHO Regional Office for the Eastern Mediterranean (2021), World Health Organization Western Pacific Region (2021), WHO Regional Office for South East Asia (2021).

b Central Intelligence Agency (2021).

c The World Bank (2021d).

d Atlas method, current US$ (The World Bank, 2021c).

e ‘A Gini index of 0 represents perfect equality, while a Gini index of 100 implies perfect inequality’ (The World Bank, 2021b).

f Political participation is a composite measure including ‘voter participation’, minority groups having autonomy and voice in the political process, ‘membership of political parties and political NGOs’, ‘citizen engagement with politics’, and ‘preparedness of the population to take part in lawful demonstrations’ (The Economist Intelligence Unit, 2020).

g Closed autocracy: ‘No multiparty elections for the chief executive or the legislature’; electoral autocracy: ‘Electoral autocracy: De-jure multiparty elections for the chief executive and the legislature, but failing to achieve that elections are free and fair, or de-facto multiparty, or a minimum level of Dahl’s institutional prerequisites of polyarchy’; electoral democracy: ‘De-facto free and fair multiparty elections and a minimum level of Dahl’s institutional prerequisites for polyarchy… but either access to justice, or transparent law enforcement, or liberal principles of respect for personal liberties, rule of law, and judicial as well as legislative constraints on the executive not satisfied’; liberal democracy: ‘De-facto free and fair multiparty elections and a minimum level of Dahl’s institutional prerequisites for polyarchy… are guaranteed as well as access to justice, transparent law enforcement and the liberal principles of respect for personal liberties, rule of law, and judicial as well as legislative constraints on the executive satisfied’ (Coppedge *et al.*, 2020a,b).

h % of total health expenditure (The World Bank, 2021a).

#### Health context and framing

It was apparent from national policy documents and reporting in the media that the taxes were being implemented in a context of government recognition of NCDs as a policy problem. NCDs were identified as a specific priority in all countries’ national health policy documents and media, as well as within the national strategy documents of 13 countries. Additionally, in the media of six countries (Brunei, India, Ireland, South Africa, Thailand and UK), tooth decay was mentioned as a health problem associated with SSB consumption.

All countries in our sample had excise (or similar) taxes to address tobacco and/or alcohol at the time of adoption of the SSB tax. Bermuda, Brunei and India had taxes on other foods, e.g. food products containing sugar, fats and cocoa. In the majority of countries (*n* = 11), the tax was discussed in the media in relation to other policy measures to address diet-related NCDs. For example, it was identified as part of a package of interventions by government actors in Bermuda, Brunei, India, Peru, Saudi Arabia, UAE and UK.

#### Economic context and framing

The adoption of an SSB tax coincided with reform of the taxation system in 11 countries, evident from the media and policy documents (Table 3). In all countries, we found an indication of economic considerations related to the adoption of the tax in the media and fiscal priorities stated in the national strategy documents (Table 3). For example, in the Arab Gulf States, who are members of the Gulf Cooperation Council (GCC), we found mention in policy documents and media, of (insufficient) revenue as a problem needing to be addressed, and the financial priorities of each government were to raise and diversify (from non-oil) revenues. In 12 of the countries, we observed explicit recognition of health as important to economic (development) goals in the national strategic documents and/or national health policy documents. In these countries, we noted that government health expenditure, as a percentage of total health expenditure, was between 51% and 95% (Table 2). Concern regarding the health-care cost of NCDs was identified in the media in half of the countries in the sample.

**Table 3.** Characteristics of case studies, organized by the theoretical framework

Country	The innovation	Diffusion networks	Change agents	Innovator countries
Indicators	Framing of the SSB tax (pre-implementation)	Reference to multilateral institutions; reporting of other countries’ SSB taxes	Type of actors and mechanisms for engagement in the policy process	Framing of the problem; fiscal priorities of government
Sources	Newspapers and news websites Policy documents Government websites	Newspapers and news websites	Newspapers and news websites	Newspapers and news websites Policy documents Government websites
Bahrain^a^	Health: reduce obesity/NCDs; increase consumption of healthier beverages; ineffective for health. Economic: health system savings, raising revenue and enhancing tax system; negative impact on food industry; regressive; incoherent with economic policy.	Reference to multilateral institutions: economic (WTO); regional institutions (GCC). Reporting of other SSB taxation: Saudi Arabia; UAE.	Supportive (government, health experts): information & messaging; constituency building; policy substitution Opposing (WTO, industry): information & messaging; constituency building; opposition fragmentation & destabilization, legal challenges.	Policy issues: obesity and/or NCDs; declining oil revenues; unhealthy food environments; high-risk population groups—females. Fiscal priorities: raising (non-oil) revenue; reducing budget deficit; economic growth; tax reform.
Bermuda^b^	Health: reduce obesity/NCDs; increase consumption of healthier beverages; ineffective for health. Economic: negative impacts on food industry; health system savings; enhancing tax system; incoherent with economic policy.	Reference to multilateral institutions: health (WHO, Multilateral Diabetes Federation). Reporting of other SSB taxation: Mexico; Finland; UK; Denmark.	Supportive (NGOs, health experts, government, consumer groups): information & messaging; policy substitution. Opposing (industry and government—opposition politicians): information & messaging; policy substitution.	Policy issues: obesity and/or NCDs; economic costs of NCDs; high-risk population groups—low income. Fiscal priorities: tax reform
Brunei^c^	Health: reduce obesity/NCDs; increase consumption of healthier beverages. Economic: not evident.	Reference to multilateral institutions: health (World Obesity Federation). Reporting of other SSB taxation: Mexico; UK; USA.	Supportive (government): information & messaging; constituency building. Opposing: non evident.	Policy issues: obesity and/or NCDs; tooth decay; high risk population groups; unhealthy food environments. Fiscal priorities: reducing budget deficit and economic growth.
India^d^	Health: reduce obesity/ NCDs; improve health of specific population groups. Economic: negative impact on food industry; raising revenue and enhancing tax system; health system savings.	Reference to multilateral institutions: health (WHO). Reporting of other SSB taxation: Mexico; France; Chile; South Africa; Philippines; Hungary; UK.	Supportive (coalition, NGOs, government, health experts, WHO): information & messaging; constituency building. Opposing (industry): information & messaging; constituency building.	Policy issues: obesity and/or NCDs; tooth decay; high-risk population groups—the poor and children. Fiscal priorities: raising revenue raising, reducing budget deficit, economic growth; tax reform.
Ireland^e^	Health: reduce obesity/NCDs; reduce tooth decay; improve health of specific population groups—children; ineffective for health. Economic: raising revenue; negative impact on food industry; regressive; incoherent with economic policy.	Reference to multilateral institutions: health (WHO). Reporting of other SSB taxation: France; Hungary; Belgium; Finland; UK.	Supportive (government—health, WHO, NGOs): information & messaging; constituency building . Opposing (government—finance, industry): information & messaging; legal challenges; constituency building.	Policy issues: obesity and/or NCDs; tooth decay; high-risk population groups—children and low income. Fiscal priorities: raising revenue; reducing budget deficit; economic growth; tax reform.
Malaysia^f^	Health: reduce obesity/NCDs; increase consumption of healthier beverages; improve health of specific population groups—children and youth; ineffective for health. Economic: raising revenue and enhancing tax system; health system savings; negative impacts on food industry; regressive; incoherent with economic policy.	Reference to multilateral institutions: health (WHO); other (UNICEF). Reporting of other SSB taxation: Finland; Greece; Vietnam; Indonesia; UK; France; US; Hungary; Mexico; Brunei; Saudi Arabia; Thailand; Chile; UAE; Norway; Philippines; India; South Africa.	Supportive (government, WHO, UNICEF, health experts): information & messaging; constituency building. Opposing (consumers, economic experts, industry): information & messaging; policy substitution.	Policy issues: obesity and/or NCDs; high-risk population groups—children, students and high income; economic cost of NCDs; unhealthy food environments. Fiscal priorities: raising revenue raising; economic growth.
Peru^g^	Health: reduce obesity/NCDs. Economic: health system savings.	Reference to multilateral institutions: health (WHO). Reporting of other SSB taxation: Chile; Ecuador; Mexico.	Supportive (government, consumers): legal challenges. Opposing (industry): information & messaging; constituency building; policy substitution.	Policy issues: obesity and/or NCDs; high-risk population groups—children, high-income; economic cost of NCDs. Fiscal priorities: economic growth; tax reform.
Philippines^h^	Health: reduce obesity and NCDs; ineffective for health. Economic: raising revenue; health system savings; negative impacts on food industry; regressive; incoherent with economic policy.	Reference to multilateral institutions: health (WHO); economic (IMF, OECD). Reporting of other SSB taxation: Denmark, Zambia, Indonesia, Mexico; Egypt, UK, Singapore.	Supportive (government, health experts, WHO, IMF): information & messaging; constituency building. Opposing (industry, NGOs, government, economic experts, consumers): information & messaging; policy substitution; opposition fragmentation and destabilization.	Policy issues: obesity and/or NCDs; high-risk population groups—low income and youth; economic cost of NCDs. Fiscal priorities: raising revenue; reducing budget deficit; economic growth; tax reform.
Portugal^i^	Health: none evident preimplementation. Economic: negative impact on food industry.	Reference to multilateral institutions: none evident. Reporting of other SSB taxation: none evident.	Supportive: none evident Opposing (industry): legal challenges; policy substitution.	Policy issues: obesity and/or NCDs Fiscal priorities: economic growth; reducing budget deficit.
Qatar^j^	Health: reduce obesity/NCDs. Economic framing: raising revenue and enhance tax system.	Reference to multilateral institutions: economic (IMF); regional institutions (GCC). Reporting of other SSB taxation: none evident.	Supportive (experts, consumers): information & messaging. Opposing: none evident.	Policy issues: obesity and/or NCDs; unhealthy food environments. Fiscal priorities: economic growth; raising (non-oil) revenue; tax reform.
Saudi Arabia^k^	Health: reduce obesity/NCDs; improve health for specific population groups. Economic: raising revenue and enhancing tax system; health system savings; regressive.	Reference to multilateral institutions: health (WHO); economic (WTO, IMF); regional institutions (GCC). Reporting of other SSB taxation: GCC countries.	Supportive (government, experts): information & messaging; constituency building. Opposing (experts, WTO): information & messaging; legal challenges; policy substitution.	Policy issues: obesity and/or NCDs; high-risk population groups—children; unhealthy food environments; economic cost of NCDs; declining oil revenues; budget deficit. Fiscal priorities: reducing budget deficit; raising (non-oil) revenue; economic growth; tax reform.
Seychelles^l^	Health: reduce obesity/NCDs. Economic: none evident pre-implementation.	Reference to multilateral institutions: health (WHO). Reporting of other SSB taxation: Mexico.	Supportive: none evident. Opposing: none evident.	Policy issues: obesity and/or NCDs; high-risk population groups—children. Fiscal priorities: reducing budget deficit; tax reform; economic growth.
South Africa^m^	Health: reduce obesity/NCDs; ineffective for health. Economic: raising revenue: health system savings; negative impacts on food industry; cost-effective policy.	Reference to multilateral institutions: health (WHO). Reporting of other SSB taxation: France; Mexico; Brazil; Hungary; Denmark; Scandinavian countries.	Supportive (health experts, coalition, NGOs, WHO): information & messaging. Opposing (industry, health experts): opposition fragmentation and destabilization; legal challenges; policy substitution; information & messaging; constituency building.	Policy issues: obesity and/or NCDs; tooth decay; economic cost of NCDs; unhealthy food environments. Fiscal priorities: raising revenue; economic growth.
Thailand^n^	Health: reduce obesity/NCDs; increase consumption of healthier beverages; ineffective for health. Economic: health system savings, raising revenue; negative impact on food industry.	Reference to multilateral institutions: health (WHO); economic; regional institutions. Reporting of other SSB taxation: USA; Mexico; France; Ecuador; Singapore; Western Pacific Island countries; Scandinavian countries; Finland; UK.	Supportive (government, NGOs, health experts): information & messaging; policy substitution; constituency building. Opposing (industry): information & messaging; policy substitution; constituency building.	Policy issues: obesity and/or NCDs; tooth decay; economic cost of NCDs; high-risk population groups. Fiscal priorities: tax reform; economic growth.
United Arab Emirates^o^	Health: reduce obesity/NCDs; ineffective for health. Economic: raising revenue and enhancing tax system; negative impact on food industry.	Reference to multilateral institutions: health (WHO); economic (WTO); regional institutions (GCC). Reporting of other SSB taxation: Saudi Arabia; Bahrain; GCC countries.	Supportive (government, experts, influencers): information and messaging. Opposing (industry, WTO): information & messaging; constituency building, policy substitution; legal challenges.	Policy issues: obesity and/or NCDs; tooth decay; economic cost of NCDs; unhealthy food environments; high-risk population groups. Fiscal priorities: raising (non-oil) revenue; economic growth; tax reform.
United Kingdom^p^	Health: reduce obesity/NCDs; improving health for specific populations—children; ineffective for health. Economic: health system savings; raising revenue; negative impacts on food industry; incoherent with economic policy.	Reference to multilateral institutions: none evident. Reporting of other SSB taxation: Denmark; Belgium; Chile; France; Norway; Hungary; Mexico.	Supportive (government, health experts, influencers, NGOs and consumers): information & messaging; constituency building; opposition fragmentation and destabilization. Opposing (industry, government): opposition fragmentation and destabilization; legal challenges; information & messaging.	Policy issues: obesity and/or NCDs; tooth decay; economic cost of NCDs; unhealthy food environments; high-risk population groups—children and low-income groups. Fiscal priorities: economic growth; tax reform; reducing budget deficit.

Sources:

a Ministry of Health (2015a), Kingdom of Bahrain (2019; 2008), 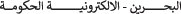  [Electronic Government—Bahrain] (٦/٣/٢٠٢١ [n.d.]).

b Ministry of Health Seniors and Environment (2016), Government of Bermuda (2018b).

c Ministry of Health (2009), Ministry of Finance (2017a), Sekretariat Tetap Wawasan Brunei 2035 [Vision of Brunei 2035 Secretariat] (2020).

d Planning Commission (2013), Ministry of Health and Family Welfare (2017), Goods and Services Tax Council (n.d.).

e Department of Health (2013), Programme for Government Office (2016), Department of Finance (2017a; 2018).

f Economic Planning Unit—Prime Minister’s Department (2015), Jabatan Kastam Diraja Malaysia [Royal Malaysian Customs Department] (n.d.), Bahagian Perancangan [Planning Unit] Kementerian Kesihatan Malaysia [Ministry of Health Malaysia] (2016).

g Gobierno del Perú [Government of Peru] (2016), Ministerio de Salud [Ministry of Health] (2019).

h Department of Finance (2017b), National Economic and Development Authority (2017), Department Of Health (2018).

i Republic of Portugal (2012; 2016).

j General Secretariat for Development Planning (2008), Ministry of Development Planning and Statistics (2018), Ministry of Public Health (2018), General Tax Authority (2019a).

k Kingdom of Saudi Arabia (2016), General Authority of Zakat and Tax (2016), General Authority of Zakat and Tax (n.d.), 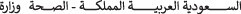. [Ministry of Health—Kingdom of Saudi Arabia], ١٦/٣/٢٠٢١ ([n.d.-a]).

l Ministry of Health (2015b), Department of Economic Planning (2019).

m National Planning Commission (2012), Department of Health (2015), South African Revenue Service (n.d.).

n National Economic and Social Development Board (2011), Bureau of Policy and Strategy (2012).

o Prime Ministers Office (2010), United Arab Emirates (n.d.), 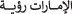  2021-  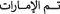  [UAE Vision 2021—United Arab Emirates], 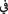 ١٦/٣/٢٠٢١ ([n.d.]).

p Conservative Party (2017), Healthier Scotland (2016), Department of Health (2013; 2016); Her Majesty’s Revenue and Customs (2016).

### Characteristics of the innovation: SSB taxation

#### Tax types

The majority of countries (*n* = 11) used an excise tax type. Furthermore, the UK and South Africa used a similar ‘levy’ approach. The excise taxes use two mechanisms—ad valorem and specific (Table 4). Ad valorem excise taxes were present in the four GCC countries (Bahrain, Qatar, Saudi Arabia and UAE), which were all HICs. Specific excise taxes, based on sugar content, were adopted in Brunei, Ireland, Malaysia, Portugal, Seychelles and Thailand (Table 4). Conversely, the Philippines adopted a specific excise tax, but based on volume and is the only lower MIC in the sample with an excise tax. Other approaches were a tariff (Bermuda), sales tax (Peru) and a value-added tax (India).

**Table 4.** Details of sugar-sweetened beverage taxes

Country	Tax type (name) and mechanism	Tax base	Tax rates
Bahrain^a^	Excise tax: ad valorem	Carbonated non-alcoholic beverages (sugar sweetened, unsweetened, other sweetener); Energy drinks; Substances intended for preparation	50% for carbonated non-alcoholic beverages; 100% for energy drinks
Bermuda^b^	Tariff: ad valorem	Non-alcoholic beverages (unsweetened, sugar sweetened or other sweeteners); Substances intended for preparation	50% for sugar sweetened beverages; 15% for other sweetened beverages; 15% for unsweetened beverages; 50% for substances intended for preparation
Brunei^c^	Excise tax: specific (sugar content)	Non-alcoholic beverages (sugar sweetened, other sweeteners)	0.40 Brunei dollars/l for: SSB with >6 g of total sugar/100 ml; soya milk drinks with >7 g of total sugar/100 ml; malt or chocolate drinks with >8 g of total sugar/100 ml; coffee-based or flavoured drinks with 6 g of total sugar/100 ml.
India^d^	Goods and Services Tax: ad valorem	Non-alcoholic beverages (unsweetened, sugar sweetened and other sweeteners); Substances intended for preparation; Fruit juices; Milk products (sugar sweetened or other sweeteners); Soya milk drinks	28% non-alcoholic beverages (sugar sweetened and other sweeteners) 28% substances intended for preparation; 18% non-alcoholic beverages (unsweetened); 12% fruit juice and soya-milk drinks; 5% milk products
Ireland^e^	Excise tax (Sugar Sweetened Drinks Tax): specific (sugar content)	Non-alcoholic beverages (sugar sweetened); Substances intended for preparation	16.26 €/hectolitre for 5–8 g sugar/100 ml; 24.39 €/hectolitre for >8 g sugar/100 ml
Malaysia^f^	Excise tax: specific (sugar content)	Non-alcoholic beverages (sugar sweetened, other sweeteners); Flavoured milk beverages (sugar sweetened, other sweeteners); Fruit and vegetable juices (sugar sweetened, unsweetened, other sweetener)	RM0.40/l for: non-alcoholic beverages with >5 g sugar/100 ml; fruit and vegetable juices with >12 g sugar/100 ml; flavoured milk beverages with >7 g sugar/100 ml
Peru^g^	Sales tax: ad valorem	Non-alcoholic beverages (sugar sweetened or other sweetener)	17% for <6 g sugar/100 ml 25% for >6 g sugar/100 ml
Philippines^h^	Excise tax: specific (volumetric)	Non-alcoholic beverages (sugar sweetened, other sweeteners including high fructose corn syrup); Energy drinks; Substances intended for preparation	6 pesos/l (sugar sweetened and other sweeteners); 12 pesos/l (high-fructose corn syrup)
Portugal^i^	Excise tax: specific (sugar content)	Non-alcoholic beverages (sugar sweetened or other sweeteners); Substances intended for preparation	8.22€/hectolitre for <80 g sugar/l 16.46€/hectolitre for ≥80 g sugar/l
Qatar^j^	Excise tax: ad valorem	Carbonated non-alcoholic beverages (sugar sweetened and other sweeteners); Energy drinks; Substances intended for preparation	50% for carbonated non-alcoholic beverages; 100% for energy drinks
Saudi Arabia^k^	Excise tax: ad valorem	Carbonated non-alcoholic beverages (sugar sweetened and other sweeteners); Energy drinks; Substances intended for preparation	50% for carbonated non-alcoholic beverages; 100% for energy drinks
Seychelles^l^	Excise Tax (Imposition of Sugar Tax on Drinks): specific (sugar content)	Non-alcoholic beverages (sugar sweetened, unsweetened and other sweeteners) Fruit and vegetable juices (sugar sweetened, unsweetened and other sweeteners)	SR4/l for >5 g of sugar/100 ml
South Africa^m^	Health Promotion Levy: specific (sugar content)	Non-alcoholic beverages (sugar sweetened, other sweeteners); Substances intended for preparation	2.1 cents/g for >4 g of sugar/100 ml
Thailand^n^	Excise tax: specific (sugar content and ad valorem)	Non-alcoholic beverages, including coffee and tea drinks (unsweetened, sugar sweetened and other sweeteners); Coffee and tea drinks (unsweetened, sugar sweetened and other sweeteners); Fruit and vegetable juices (unsweetened, sugar sweetened and other sweeteners); Substances intended for preparation	14% for non-alcoholic beverages 10% for fruit and vegetable juices PLUS: Non-alcoholic beverages: 0.10 Baht for 6–8 g sugar or other sweeteners/100 ml 0.30 Baht for 8–10 g sugar or other sweeteners/100 ml 0.50 Baht for 10–14 g sugar or other sweeteners/100 mL 1.00 = 14 g+ sugar or other sweeteners/100 ml Fruit and vegetable juices: 0.10 Baht for 6–8 g sugar or other sweeteners/100 ml 0.30 Baht = 8–10 g sugar or other sweeteners /100 mL 0.50 Baht = 10–14 g sugar or other sweeteners/100 ml 1.00 = 14 g+ sugar or other sweeteners/100 ml Substances intended for preparation: 9.00 Baht = 0–6 g sugar or other sweeteners/100 ml 10.00 Baht = 6–8 g sugar or other sweeteners/100 ml 12.00 Baht = 8–10 g sugar or other sweeteners/100 ml 13.00 Baht = 10–14 g sugar or other sweeteners/100 ml 16.00 = 14 g+ sugar or other sweeteners/100 ml
United Arab Emirates^o^	Excise tax: ad valorem	Carbonated non-alcoholic beverages (sugar sweetened and other sweeteners); Energy drinks; Substances intended for preparation	50% for carbonated non-alcoholic beverages; 100% for energy drinks
United Kingdom^p^	Soft Drinks Industry Levy: specific (sugar content)	Non-alcoholic beverages (sugar sweetened and unsweetened)	18p for 5–8 g of total sugar/100 ml; 24p for >8 g of total sugar/100 ml

Sources:

a Kingdom of Bahrain (2019), 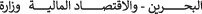  [Ministry of Finance and National Economy—Bahrain], 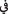 ١٦/٣/٢٠٢١. ([n.d.]).

b Government of Bermuda (2018a,b).

c Ministry of Finance (2017a,b), Jabatan Kastam dan Eksais Diraja [Royal Excise and Customs Department] (n.d.).

d Ministry of Finance (2017c), Central Board of Indirect Taxes and Customs (2019), Goods and Services Tax Council (n.d.).

e Office of the attorney General (2017), Revenue (2020).

f Jabatan Kastam Diraja Malaysia [Royal Malaysian Customs Department] (2018; n.d.).

g Ministerio de Economía y Finanza [Ministry of Economy and Finance] (2018).

h Congress of the Philippines (2017), Department of Finance -Tax Reform (2019).

i Diário da República Eletronico (2020).

j General Tax Authority (2019a,b).

k General Authority of Zakat and Tax (2016; n.d.-a,b).

l Minister of Finance Trade Investment and Economic Planning (2019), Seychelles Revenue Commission (2019).

m Republic of South Africa (2017), South African Revenue Service (2018; n.d.).

n Osornprasop *et al.* (2018), 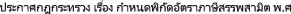  [Ministry of Finance Notification on Excise Tariff B.E. 2560 (2017)], 2560 ([2017]).

o President of the United Arab Emirates (2017), Federal Tax Authority (2019), United Arab Emirates (n.d.).

p Her Majesty’s Revenue and Customs (2016; 2018a,b).

#### Tax rates, base and object

The tax rates varied across countries, with ad valorem tax rates on non-alcoholic beverages ranging from 14% (Thailand) to 50% (Bermuda and the GCC countries) (Table 4). Rates specifically for fruit and/or vegetable juices were in the range of 10% (Table 4). The GCC countries had the highest tax rates in the sample, with 100% on energy drinks (Table 4). Five countries, using a specific tax mechanism, had tiered tax rates with minimum thresholds of grams of sugar, and the Philippines applied higher rates for beverages sweetened with high-fructose corn syrup compared to sugar (Table 4). Four of these five countries had a policy objective related to product reformulation.

The object of taxation varied across the countries; some focussed solely on sugar-sweetened non-alcoholic beverages, and others were inclusive of a broader range of beverages and types of sweeteners. All countries taxed beverages within the broad category of ‘non-alcoholic beverages’, with Ireland and the UK having the narrowest tax base (only sugar-sweetened non-alcoholic beverages) (Table 4). Fruit and/or vegetable juices were included in the tax base of only five countries, and milk beverages (sugar-sweetened) were included in the tax base of three countries (Brunei, India and Malaysia). In 14 countries, the object of the tax included unsweetened beverages and/or beverages sweetened with high-fructose corn syrups and artificial sweeteners, in addition to SSBs (Table 4). The majority of countries also included ‘substances intended for preparation’, such as powder or liquid concentrates (Table 4).

#### SSB taxes as a ‘policy solution’

In the lead up to the adoption of the taxes in all countries, there were consistent health and economic frames evident in the sampled media regarding the SSB tax as a ‘policy solution’. The consistent health frame was that the tax would be an effective means to reduce obesity and/or NCDs and was evident in statements by government, health and economic experts, and non-government actors, as well as consumer groups in Peru (Table 3). In two countries (Ireland and Thailand), we also found an indication in the media that the tax was a solution to address poor dental health. In all countries, except Brunei and Peru, taxes were framed in our media sample as effective means to raising revenue, either general revenue or earmarked for health or social purposes, to justify their adoption (Table 3).

Prior to adoption, the likely impact of the tax was also described using consistent (negative) frames in the sampled media in most countries, predominantly by food and beverage industry actors. These included the tax being ineffective for health and the tax resulting in economic impact on the food industry (Table 3). There was evidence of concerns related to regressivity of the SSB tax (i.e. a greater economic impact on the poor) in the media of seven countries by a variety of actors, including government, non-government, industry and consumer groups (Table 3).

This differential framing of the tax continued in the year following adoption. There was evidence in the media in nine countries that industry actors continued to lobby against the tax, in some cases with alternate frames. The most common negative frames post-adoption included the following: economic impact on food industry (i.e. declines in company revenue); tax compliance and/or collection issues; tax would be ineffective for health; and disagreement on the tax base. Alternate frames post-adoption were evident in Malaysia, Portugal, Thailand and UK, were there was evidence of framing of the tax having minimal economic impact on the food industry, which was inconsistent with the industry actors framing of the economic impact of the tax in the pre-adoption period, outlined above.

### Diffusion networks and policy learning

#### Reference to other countries’ SSB taxes

We found reference to SSB taxes adopted by other national governments in the sampled media of all countries, except Portugal (Table 3). Mexico was reported in the media of 10 countries and in formal policy documents in the Philippines (Department of Finance, 2017a) and South Africa (National Treasury, 2016). Notably, the countries referencing Mexico did not adopt the same tax and instead utilized a variety of tax types, bases and rates (Table 4). The mentions of Mexico’s tax were largely positive, however, in Bermuda, the Philippines and South Africa, reference to Mexico’s tax was also made by actors who were against the tax. In the GCC countries, the references to taxes elsewhere were only to other countries within the GCC.

#### Regional influence

The references to other countries’ SSB taxes in the sampled media appeared to indicate a preference for countries in the same region or membership of the same regional economic bodies. The GCC acted as a diffusion network within the Arab States of the Gulf as a result of the adoption of a Common Excise Tax Agreement (Gulf Cooperation Council, 2016) in November 2016. Reference to countries with membership of common economic groupings was also evident. In India and South Africa, there was reporting of other BRICS countries (Brazil, Russia, India, China and South Africa), in relation to similar policy problems (namely obesity) and adoption of similar tax measures. Similarly, in Ireland and the UK, reference was made to other countries in the European Union (EU); in Malaysia of taxes of other members of the Association of Southeast Asian Nations (ASEAN); and in Peru of taxes of other members of Free Trade Area of the Americas (FTAA) (Table 3). Furthermore, we noted reports of taxes in countries with shared borders, including reporting of the UK tax in Ireland; taxes in Thailand and Brunei in Malaysia; and taxes in Ecuador and Chile in Peru (Table 3).

#### Reference to multilateral institutions

We found reference to WHO in the media as an expert body, or source of recommendations regarding diet-related fiscal policy in the sampled media in 11 countries, which were all of the lower and upper MICs and half of the HICs in the sample. In five of these countries, there was an indication in the media that the WHO made public statements of support for the tax (Table 3). In contrast, we found fewer references to economic institutions. The International Monetary Fund (IMF) was evident in the media in the Philippines, Qatar and Saudi Arabia and the World Trade Organization (WTO) in the media of three of the GCC countries (Bahrain, Saudi Arabia and UAE) (Table 3).

### Change agents

It was evident in the media across all countries that there were policy actors actively supporting (‘for’) and opposing (‘against’) the SSB taxes. In eight countries, we found an indication of the Ministry of Health actively supporting the policy. In four of these countries (Brunei, Malaysia, Philippines and Thailand), as well as Peru, the finance sector of government publicly supported the tax (Table 3). The most common mechanisms for engagement with the policy process used by government policy actors to support the taxes were information and messaging (*n* = 10) and constituency-building (*n* = 7) (Table 3). There was no evident association between evidence of active government support for the tax and the proportion of health expenditure borne by government in these countries or the prevalence of related health conditions (Table 2, Figure 2). In five countries, opposition to the tax from government actors was also evident in the media. For example, in the Philippines, Leftists House members opposed the tax, and in the UK, the Health Secretary and Prime Minister (in the post-adoption period) opposed the tax.

Food and beverage industry actors, including both local and multinational industries, acted as policy change agents ‘against’ the tax, in all but one country in which opposition to the tax was evident in the media (Table 3). Local industry actors against the tax were evident in the media of half of the countries. Multinational corporations, or local subsidiaries, were evident in the media against the tax in 10 countries. Industry actors spanned the food system including the following: SSB manufacturer and distributors in over half of the countries; food and/or beverage industry associations in half the countries; sugar industry associations (India, South Africa); dairy manufacturer and wholesaler (Bermuda); and retailers (Philippines). The use of constituency-building and information and messaging approaches to lobby against the tax were evident in the media in all countries in which actors ‘against’ SSB taxation were evident (*n* = 14). Change agents ‘against’ the tax used mechanisms including legal challenges (*n* = 7) and policy substitution (i.e. proposing other policies) (*n* = 9), compared to *n* = 1 and *n* = 3, respectively, by actors supportive of SSB taxation. Other non-government actors opposing the tax identified in the media included the WTO (Bahrain, Saudi Arabia and UAE); consumer groups (Malaysia and Philippines); economic or health experts (Malaysia, Philippines and South Africa); and non-government organizations (NGOs) (Philippines).

Countries in which we saw evidence in the media of active industry engagement ‘against’ the tax also had a large presence of policy change agents ‘for’ the tax. For example, India had a coalition of health experts; Ireland had bipartisan support; Philippines had support from the Department of Health and Finance, as well as the WHO; South Africa had support from a coalition of health experts and NGOs; and UK had support from health experts, a coalition of NGOs and government actors. In most countries (*n* = 13), we found evidence of support for the tax from stakeholders outside of government in the media sample, including NGOs, medical, public health and economic experts, and consumer groups.

## Discussion

This study analysed the characteristics and patterns relevant to SSB tax adoption as a cross-sectoral policy ‘innovation’ in 16 countries. Overall, we found significant adaptation and heterogeneity in the approaches used for SSB taxation (‘innovation’) and in the characteristics of the ‘innovating countries’. In line with previous research (Hagenaars *et al.*, 2017; Graça *et al.*, 2018; Onagan *et al.*, 2019), we found consistency in the framing of the health problem—particularly with reference to obesity and/or NCDs as major health problems—and in the economic considerations, such as the need to raise revenue, health being fundamental for development, and the rising economic health-care cost of NCDs. Two key points at which this study has extended previous work is in the comparative consideration of actor influence and the patterns of policy learning across ‘diffusion networks’.

The majority of countries adopted excise taxes, in line with expert recommendations regarding tax type (WHO, 2016). However, countries adapted the innovation to be relevant to their context. Consistent with previous findings regarding the importance of institutional capacity for tax design (James *et al.*, 2020; Fuster *et al.*, 2020), we found specific excise taxes on nutrient content in UMICs and HICs and a simpler specific excise tax based on volume adopted in the Philippines (a LMIC). A tiered tax rate structure was utilized by countries to align with policy objectives to promote product reformulation by beverage manufacturers. These tax designs have had industry support, as there is a economic benefit to beverage manufacturers to reformulate (Alsukait *et al.*, 2020a). Adaptation of the global recommendations was also evident in the tax object and rate. There is a need for future research to examine the extent to which adaptation reflects industry opposition.

One benefit of examining SSB taxation as a policy ‘innovation’ with a focus on policy learning was the insights gained regarding reference points and learning for SSB taxes. We found reference to taxes in other countries, particularly those with evaluations (namely Mexico), those with shared membership of economic bodies and those with shared borders (due to issues of tax compliance). Notably, the GCC acted as a regional diffusion network for taxes within the Arab Gulf States (Alsukait *et al.*, 2020a). For these countries, the GCC can also be considered a change agent as the Gulf Health Council, part of the GCC General Secretariat, is leading the implementation of a Unified Gulf Plan for Control of Noncommunicable Diseases (Gulf Health Council, 2020). Among all the countries in our sample, multilateral health institutions (i.e. WHO) were more likely to act as diffusion networks than economic institutions (i.e. IMF), particularly for MICs. However, there is potential for economic institutions to also foster diffusion of SSB taxes. For example, diffusion among the Arab Gulf States was associated with objectives of ‘economic integration’ and ‘to promote the GCC economy’ (Gulf Cooperation Council, 2016). Our findings regarding the role of change agents and diffusion networks in fostering uptake of SSB taxes reflect work by Gautier and others on the importance of both national and transnational stakeholders in health policy diffusion (see, e.g., Gautier (2021) and Gautier et al. (2018)). We thus extend this important work into the field of nutrition policy.

Active support from government actors was a common feature in our case study countries. This reflects previous findings that high-level government commitment, especially by ‘veto players’, i.e. those who have to agree for policy change to happen (Fuster *et al.*, 2020), has been an enabling factor for SSB adoption in France, Mexico and the Philippines (Onagan *et al.*, 2019; Le Bodo *et al.*, 2019; Fuster *et al.*, 2020). However, we also found active public debate and lobbying regarding SSB taxation in all countries. Food and beverage industry actors were the most common actor opposing the SSB taxes, raising concerns that taxes would be ineffective for health and have economic impacts on the food industry and the poor (due to regressivity of the tax). This is consistent with the findings of in-depth studies in Mexico and elsewhere, which have found public opposition by the SSB industry (Ojeda *et al.*, 2020; Carriedo *et al.*, 2021). It is notable that industry opposition on the basis of economic impacts occurred despite evidence that these are groundless (Mounsey *et al.*, 2020; Law *et al.*, 2020). In countries where there was both opposition and public consultation on the tax, we found networks of policy actors supporting the tax, including individuals and coalitions of health, and economic experts and NGOs.

This study presents a comprehensive analysis of SSB taxes in 16 countries, drawing on qualitative approaches for documentary research, but has three main limitations. First, the use of policy and media data sources may only provide a partial perspective on the policy process and experience in the 16 case study countries. The sampling strategy for the media articles, the focus on newspaper articles and a lack of assessment of potential bias in the media sources may have resulted in incomplete or partiality in the data. Furthermore, the lack of human resources and language skills in Portugal, Seychelles and the Philippines may have inadvertently resulted in inaccuracy in the data in these case study countries. However, we were able to conduct data analysis in at least one official language other than English in eight countries. Second, within our study frameworks, we extended the application of corporate political activity strategies to non-corporate actors, including government and non-government actors, which may have resulted in other mechanisms of engagement used by non-corporate actors being omitted from the study. We found that this accurately represented the types of strategies in our data (although the full range was not evidently used by non-corporate actors). Finally, the taxes were only analysed at the point of adoption and for 1 year post adoption in the media. Thus, subsequent changes to the tax bases and rates which have occurred in countries (e.g. Bahrain, UAE, Saudi Arabia, Qatar, Portugal and Thailand) were not included. As these taxes continue to evolve, further research into influences on subsequent changes will be valuable for informing the ongoing strengthening of taxation.

### Lessons for global policy adoption

The findings of this study point to three lessons for future adoption of SSB taxes. First, in line with previous research, tax system reform appears to offer a policy window for SSB taxation (Onagan *et al.*, 2019; Le Bodo *et al.*, 2019; Alsukait *et al.*, 2020a). In the Philippines and India, SSB taxes were introduced as part of widespread tax reform. Further, significant budgetary pressure and enhancements to the tax system can also provide a window for adoption. For example, the Mexican government’s need to raise revenues for public expenditure was a key driver for the soda tax adoption (Carriedo *et al.*, 2020), and the adoption of an SSB tax by France in August 2011 was influenced by budgetary deficits and a government priority to reduce the economic burden of public health insurance, following the Global Finance Crisis during the late 2000s (Le Bodo *et al.*, 2019). Similarly, the current post-COVID-19 global economy could create a policy window for SSB tax adoption, as countries look for opportunities to raise revenue for vital social services with minimal social and economic impacts (Marquez and Dutta 2020). SSB taxes could thus present a ‘triple-win’ policy action due to the positive impacts on revenue, NCDs and coronavirus disease 2019 (COVID-19) (resulting from the established link between obesity/NCDs and COVID-19 outcomes) (Popkin *et al.*, 2020).

Second, actors who would like to support SSB tax adoption need to prepare for strong and public opposition, which has the potential to delay or weaken taxation. Industry opposition in Chile and Mexico (two ‘early adopter’ countries) resulted in sub-optimal tax rates (Fuster *et al.*, 2020). In Mexico, civil society organizations acted as knowledge brokers between experts and policymakers and strategically engaged with the policy process through public demonstrations and media campaigns (Fuster *et al.*, 2020; Carriedo *et al.*, 2020). Other avenues for influence identified through our study include engaging with public consultations and referencing other countries that have achieved ‘success’ in terms of adopting an SSB tax. Our analysis indicated that this strategic engagement with policymakers by health actors needs to continue beyond adoption of the tax, as public arguments opposing the taxes continued in the year following adoption, in all countries.

Finally, the experience and evidence from ‘early adopter’ countries seem to have been important in the diffusion of SSB taxes in the ‘early majority’ countries. Diffusion of innovation theory posits that ‘potential adopters look to early adopters for advice and information about an innovation’ (p. 283) and ‘serve as a role model for many other members of a system’ (p. 283) (Rogers, 2003). This highlights that evidence of effectiveness of previous SSB taxes, as well as information regarding experiences of tax design and implementation, can be helpful to policy actors seeking to adopt taxes in the future. This suggests a role for actors who play an intentional role as ‘diffusion networks’ for NCD prevention policy, such as the WHO, in fostering the compilation and dissemination of evidence and best-practice guidance for SSB taxation globally. It also suggests that in countries with successful adoption of SSB taxes, evaluation of process, impact of the taxes on consumption and health, and dissemination of findings can support future adoption globally, as seen in Mexico (Fuster *et al.*, 2020). However, our study countries also reported on taxes in other ‘early majority’ countries, which suggests that the reference point for future adopters may in fact shift from ‘early adopters’ to ‘early majority’ adopters (and so on) as global adoption of the innovation increases. This finding also suggests opportunities for future research examining the use of evaluations of ‘real-world’ policies to inform SSB tax adoption and implementation.

The findings of this study may also provide relevant insights regarding the policy processes surrounding other cross-sectoral nutrition policy interventions, including front-of-pack labelling, school food policy interventions and restrictions on marketing to children. These have been recommended by the WHO (WHO, 2013) but similarly require engagement with sectors outside of health for their operationalization at the national level and are likely to have economic impacts on industry actors (Reeve *et al.*, 2018; Dorlach and Mertenskötter 2020). For example, previous research into front of pack nutrition labelling and marketing restrictions has identified the potential for economic concerns, including potential impacts on trade and industry growth, to hamper adoption (Thow *et al.*, 2018b; 2021b). As such, the lessons identified in these case studies of SSB taxation ‘success’—particularly the frames and mechanisms used for public and political engagement, and the need for advance consideration of economic policy agendas and likely industry arguments—may also prove useful in supporting adoption of other nutrition policy initiatives and point to further opportunities for in-depth research.

## Conclusion

This comparative study of the experience of 16 countries in implementing SSB taxation found adaptation and heterogeneity in the approaches used for SSB taxation. We found consistent health and economic considerations presented by those supporting and opposing the taxes. We identified three key lessons for countries considering adoption of SSB taxes. First, engaging with the economic context is important: tax system reform appears to offer a policy window, and economic concerns formed the basis for strong opposition by industry. Second, effective health sector advocacy was characterized by consistent health frames and strategic engagement with the policy process, in some cases by cross-sector coalitions. Third, robust evaluation and reporting of SSB taxation may foster global policy learning.

## Supplementary Material

czac004_Supp

## Data Availability

The data underlying this article will be shared on reasonable request to the corresponding author.

## Endnotes

1. The World Health Organization defines SSBs as ‘beverages containing added caloric sweeteners, such as sucrose, high-fructose corn syrup, or fruit-juice concentrates. These include, but are not limited to, carbonates, fruit drinks, sports drinks, energy and vitamin water drinks, sweetened iced tea, and lemonade’ (WHO, 2016)..

2. As we began to collect data from policy documents, we were able to ascertain the exact date of the legislative decision to adopt the tax, in addition to the date of implementation (see Figure 1 for the variation between adoption and implementation dates in our sample). For reference, in this study, implementation was defined as the date the legislation came into effect, whereas adoption was the date legislation was passed. As a result, some of the later ‘early adopter’ countries are included in our sample, due to adoption occurring in the year prior to implementation. (This is unlikely to have any implications for the findings, as our study focus was on more recent adopters, rather than specific characteristics or differences between categories of adopters.)
