# Supplementary material for: A comparative policy analysis of the adoption and implementation of sugar-sweetened beverage taxes (2016–19) in 16 countries
Source: Health Policy Plan. 2022 Mar 4;37(5):543–64. doi: 10.1093/heapol/czac004 (PMC9113088; doi:10.1093/heapol/czac004)
Supplement: czac004_Supp [file czac004_supp.zip › DOI SSB Taxes_Supp_26Mar21.docx]

## SUPPLEMENTARY MATERIAL

*Media data collection*

We developed search terms with librarian support, based on the initial review of tax names and bases: ((Sugar* OR Sweeten* OR Carbonate* OR Fizzy OR Soft OR Aerate* OR Mineral OR Flavo?red OR Non-alcoholic OR concentrat*) near4 (Beverage* OR Drink* OR Juice* OR Powder* OR Gel* OR Extract*) OR "sports drink*" OR "energy drink*" OR "mineral water*" OR "flavo?r* water*" OR “flavo?r* milk*” OR Soda* OR Cola* OR Coke) AND (Tax* OR Tariff* OR Excise* OR Duty OR Duties OR Levy OR Levies) AND [Country Name]. Searches spanned a 5-year time period from 1^st^ January of 3 years prior to year of adoption, to the 31^st^ of December of 1-year post-adoption of the SSB tax, to include the agenda setting period and the initial period post adoption of the tax. The initial media searches were conducted by one researcher (author 7) on Factiva database, in English. Searches were conducted in Factiva and local news databases in the official languages of the innovator for n=8 countries, due to limited search results in English from the Factiva database.

We extracted a sample of relevant articles across the 5-year period using a targeted approach. For each country, we sampled two articles per month: the first article to meet the inclusion criteria in the first half (1^st^ to 15^th^) and second half (16^th^ to end) of each month. The inclusion criteria were the article: was published in media based in the innovator country (since we were interested in domestic rather than international framing of the taxes); and discussed SSB tax in relation to any stage of the policy process (agenda setting, policy consultation/formulation, decision making, implementation, evaluation). Articles were excluded if they were: not published in the innovator country (e.g. international newswire publications); industry publications (e.g. hospitality, beverage); discussed SSB taxes at a sub-national level in the innovator country (e.g. state taxes in India); and mentioned SSBs but not the tax or an aspect of the policy process. To validate the sampling process, two researchers (authors 1 and 7) sampled the search results from one country, which were compared, and the inclusion and exclusion criteria for the sampling approach was amended as required.

*Media data coding*

To extract data from the sampled media articles, relevant to the theoretical framework, a codebook (Table 4) was developed and tested by members of the research team [blinded for review]. Coding was conducted by three researchers [blinded for review] for all countries using NVivo 12™ and the codebook to apply descriptive codes to full sentences of the text (unless it contradicted another code) and applying multiple codes to text (Table 5). Information specific to the policy instrument were not coded as this information was collected in the documentary data.

*Media data preliminary analysis*

Three researchers [blinded for review] reviewed the data and inductively determined themes for the ‘problem’, ‘solution’ and ‘solution–other approaches’ codes, by analysing across the countries (n = 5) for which there was a substantive number of media articles in English (n = > 20). Typologies for ‘mechanisms of engagement’ by policy entrepreneurs were adapted from the framework in Mialon et al. (2015). One researcher [blinded for review] applied the themes to the data for the countries (n= 13) with media search results in English. A template outlining the themes and typologies, as well as presence of change agents and diffusion networks was developed. Four researchers [blinded for review] deductively analyzed the media data using the template for media searches conducted in Arabic, Thai, Spanish and Bahasa Melayu (n=8).

*Insert Table 5*
